# In situ Electrical Impedance Tomography for Visualizing Water Transportation in Hygroscopic Aerogels

**DOI:** 10.1002/advs.202402676

**Published:** 2024-05-14

**Authors:** Miao Tang, Haosong Zhong, Xupeng Lu, Rongliang Yang, Connie Kong Wai Lee, Yexin Pan, Yi Chen, Mitch Guijun Li

**Affiliations:** ^1^ Center for Smart Manufacturing Division of Integrative Systems and Design The Hong Kong University of Science and Technology Clear Water Bay Kowloon Hong Kong SAR 999077 China

**Keywords:** cellulose nanocrystals, electrical impedance tomography, emulsion template, moisture absorption, water diffusion

## Abstract

The global water crisis demands immediate attention, and atmospheric water harvesting (AWH) provides a viable alternative. However, studying the real‐time subtle relationship between water absorption, diffusion, and internal structure for hygroscopic materials is challenging. Herein, a dynamic visualization technique is proposed that utilizes an in situ electrical impedance tomography (EIT) system and a precise reconstruction algorithm to achieve real‐time monitoring of the water sorption process within aerogels from an internal microstructural perspective. These results can be inferred that composites' pore sizes affecting the kinetics of their moisture absorption. In addition, the diffusion path of moisture absorption and the distribution of stored moisture inside aerogels exhibit intrinsic self‐selective behavior, where the fiber skeleton of the aerogel plays a crucial role. In summary, this work proposes a generic EIT‐based technique for the in situ and dynamic monitoring of the hygroscopic process, pointing to an entirely new approach regarding research on AWH materials.

## Graphical abstract

A novel visualization technology using an in situ electrical impedance tomography (EIT) system with an accurate reconstruction algorithm can monitor the dynamic differential moisture diffusion behavior in the water sorption process, providing an effective method for studying hygroscopic behavior and in situ regulation. This work significantly simplifies atmospheric water harvesting (AWH) characterization technology and brings a new perspective to investigate hygroscopic materials.

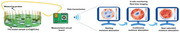

## 1 Introduction

Energy and water are essential resources for humans and have a pivotal impact on the progress of human societies.^[^ ^1^, ^2^ ^]^ Porous materials achieve water sorption from the atmosphere, which is extensively applicable to water harvesting projects and improving energy efficiency. There are several mainstream methods of water sorption. In this regard, dehumidifiers are efficient regarding their applicability in water sorption processes. However, they consume a high amount of energy.^[^ ^3^ ^]^ On the other hand, sorbent‐based materials, such as silica gel, acticarbon, and hygroscopic salts, exhibit an excellent ability to harvest atmospheric moisture,^[^ ^4^ ^]^ they show promising applicability in water collecting and dehumidification projects. Thus, studying the optimization and regulation of their hygroscopic behavior can help enhance the moisture absorption efficiency of such hygroscopic materials.

One of the key issues hindering the development of hygroscopic materials is that their hygroscopic kinetics are complex and difficult to characterize using existing techniques. Indeed, the most common among the relevant characterization methods and techniques currently used to visualize and validate dynamic hygroscopic behavior can be categorized into several types: mass measurement, molecular dynamics simulation, and some advanced structural characterizations (e.g., in situ Raman mappings, X‐ray three dimensional microscopy, and two dimensional micro FTIR).^[^ ^3^, ^5^, ^6^ ^]^ Despite their usefulness, these techniques provide intermittent measurements and do not outline the complex moisture absorption process from a holistic perspective by considering factors such as cost, scalability, sensitivity, etc. Thus, introducing novel and standardized methodologies to monitor a hygroscopic process in situ can elucidate the relationship between the properties of a material and its hygroscopic behavior, in addition to describing the material structure of sorption kinetics; such methodologies, in turn, can provide valuable guidance regarding the practical design of hygroscopic materials.

**Figure 1:**
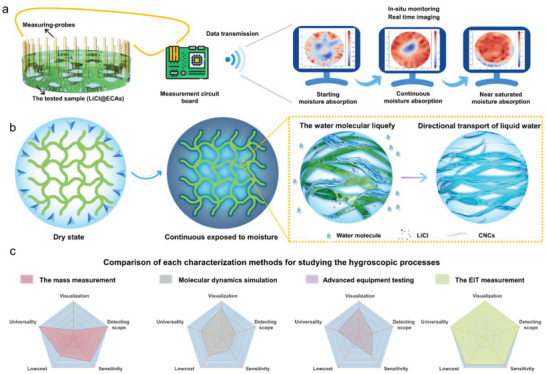
The EIT technique and design of LiCl@ECAs. a) Schematic diagram showing the devices used to reconstruct the conductivity distribution based on pillar electrodes at the boundary of the wall is used to monitor the LiCl@ECAs (we took voltage measurements at every electrode for each current injection). b) Schematic displaying the water molecular liquidation and directional transport liquid water behaviors of the LiCl@ECAs. c) The mainstream characterization methods and techniques currently used to study dynamic hygroscopic behavior can be categorized into several types: mass measurement, molecular dynamics simulation, and some advanced structural and spectral characterizations. Evolution matrices of the cross‐comparisons in terms of the degree of visualization, detecting scope, universality of samples, testing sensitivity, and costs, which exhibits the EIT system has advantages in studying hygroscopic processes in these five factors.

In this respect, electrical impedance tomography (EIT) is a non‐intrusive imaging technology that utilizes electrical measurements from boundary electrodes to map the relative conductivity distribution within a sample or targeted region.^[^ ^7^, ^8^ ^]^ By applying small electrical currents to a given sample and recording the response voltages at the sample surface, EIT reconstructs a conductivity map, providing valuable insights into the underlying structures and activities within the sample.^[^ ^9^ ^]^ This technique has found applications in various fields such as petrochemicals and biomedicine. It is commonly used to estimate the two dimensional distribution of oil, gas, and water in multi‐phase flow transportation pipelines. The electrode distribution mode has been extensively investigated and validated, based on a solid scientific foundation.^[^ ^10^, ^11^ ^]^ In this study, we have employed the conventional EIT electrode distribution mode to improve the accuracy of moisture absorption process estimation. Specifically, during the moisture absorption process, the electrical conductivities of each area of a hygroscopic material change dynamically. By analyzing the dynamic electrical impedance caused by the distribution of these dynamically changed conductivities, EIT, along with various reconstruction algorithms, can create real‐time images or dynamic reconstructions of the internal conductivity distribution of a given material, thus accurately reflecting its hygroscopic process.^[^ ^12^ ^]^ Considering these abovementioned advantages, one expects that EIT can enable the real‐time monitoring of the moisture absorption process inside hygroscopic materials (**Figure** **1**a).

To demonstrate the actual performance of the EIT‐based visualization technique regarding the hygroscopic process, a high‐performance atmospheric water harvesting (AWH) material is necessary. Fortunately, researchers have developed various methods and materials for gathering atmospheric water vapor on a global scale in recent years. Specifically, metal–organic frameworks (MOFs) demonstrate a tremendous capacity for absorbing moisture from the opening environment owing to the hydrophilicity, ultrahigh specific surface area, porosity, and chemical stability.^[^ ^13^, ^14^, ^15^, ^16^, ^17^, ^18^ ^]^ Moreover, other carbonaceous materials, such as graphene aerogels, have exhibited excellent performance for hygroscopicity owing to their porous architecture and massive reactive sites.^[^ ^19^, ^20^, ^21^ ^]^ In addition, hygroscopic gels have shown great promise in this regard; indeed, they serve as platforms facilitating high water uptake from the atmosphere due to their customizable internal structure and strong water retention capabilities.^[^ ^22^, ^23^, ^24^, ^25^, ^26^, ^27^, ^28^ ^]^ The above data reveal that an ideal absorbent material should have an ultrahigh hydrophilicity, a sustainable hygroscopic area, a self‐supporting structure, and a high‐water storage capacity.

A porous and self‐supporting biopolymers skeleton is an ideal option that can meet the abovementioned requirements. On one hand, the emulsion template method can generate internal micro‐architecture of channels and pores by utilizing emulsion droplets as the sacrificial template while removing the oil and water phases.^[^ ^29^, ^30^, ^31^ ^]^ On the other hand, nanocellulose, as an accessible, sustainable, and remarkably strong biopolymer, holds great promise as an emulsifier and can produce a wall and skeleton with continuous porosity.^[^ ^32^, ^33^, ^34^, ^35^ ^]^ Hence, combining the emulsion template method and nanocelluloses can enable the maintenance of porous structures and abundant water transport pathways inside the self‐supporting aerogels, thus providing a large number of loading sites for hygroscopic salts and increasing the moisture absorption efficiency of the given hygroscopic material. Moreover, integrating lithium chloride (LiCl) into a cellulose‐based aerogel can further elevate the latter's moisture absorption capacity and efficiency. Indeed, the LiCl particles tend to be encapsulated in the external part of the nanocellulose skeleton; a few of them can be loaded into the internal channel, as a result of which the atmosphere's moisture can also be smoothly transported from the external to the internal environment for long‐term water absorption.^[^ ^28^, ^36^ ^]^ Moreover, the “honeycomb” or the hierarchical pore structure can partly seal hygroscopic salts to decelerate the loss of LiCl.^[^ ^37^ ^]^ Owing to its solvent removal, the given aerogel can get ample internal water storage space to achieve superior and wholesale hygroscopicity.^[^ ^38^, ^39^, ^40^ ^]^

Herein, we report an in situ real‐time monitoring technology, with the EIT system at its core, that allows a qualitative study of the hygroscopic process to enable the understanding of the following question: what are the key factors contributing to a material's high‐rated hygroscopic performance? First, we fabricate cellulose‐nanocrystals‐based aerogels (LiCl@ECAs) using a simple two‐step steeping method. The emulsion droplets act as sacrificial templates, providing numerous active moisture absorption sites, optimized water transport pathways, and generous internal water storage space. Then, we achieve the in situ characterization of LiCl@ECAs’ hygroscopic processes by utilizing a dynamic image reconstruction method. The outcomes of our research reveal that pore sizes of LiCl@ECAs play a pivotal role in influencing the kinetics of extensive moisture absorption. Furthermore, the amount and direction of moisture diffusion exhibit significant intrinsic self‐selective behavior influenced by the fiber skeleton (Figure 1b). Hence, this study presents an innovative methodology for studying the moisture absorption of hygroscopic gels by utilizing an in situ EIT monitoring system, filling gaps in the understanding of dynamic hygroscopic evolution and facilitating the development high‐performance hygroscopic materials (Figure 1c).

## 2 Results and Discussion

### 2.1 Design and Construction of Hygroscopic Aerogels

It illustrates the design strategy and fabrication process regarding LiCl@ECAs (Figure S1, Supporting Information). Inspired by a report demonstrating the assembly of nanocellulose into continuous sub‐micron fibers with improved elasticity at −196 °C,^[^ ^41^ ^]^ we apply the same process to treat the cellulose nanocrystals (CNCs) in this study. First, we prepare a stable emulsion of CNCs using an ultrasonic method. In this phase, methylcellulose cross‐links on CNCs through hydrogen bonds to enhance the stability of the emulsion (Figures S2 and S3, Supporting Information).^[^ ^42^ ^]^ After that, pouring this emulsion into a mold and freeze‐drying it allows for the easy peeling‐off and the direct use of the resultant ECAs. Furthermore, the fabrication of tunable sizes and shapes of the ECAs is feasible owing to the above measure (Figure S4, Supporting Information). Notably, we choose LiCl as the active salt to be embedded within the cellulose nano‐networks due to its low dehydration temperature, lightweight, and high hygroscopicity.^[^ ^22^, ^24^ ^]^ Coming back, we immerse the ECAs in a 10 wt.% LiCl solution for 12 h to ensure the adequate presence of lithium (Li^+^) and chloride (Cl^−^) ions on the cellulose fibers. The crystal precipitation of lithium chloride around the nanocellulose networks is facilitated by freeze‐drying the cellulose fibers at −60 °C.

**Figure 2:**
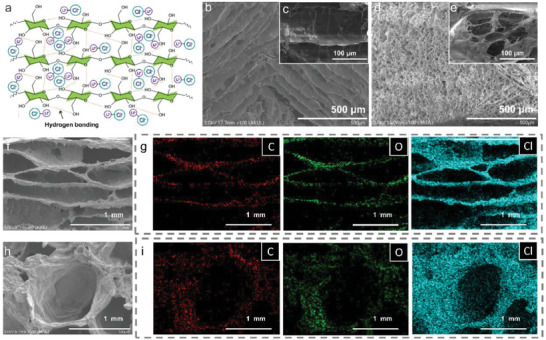
Characteristics of LiCl@ECAs. a) Schematic illustration of the interaction of LiCl ion with the CNCs’ skeleton. b,c) FE‐SEM images of ECA 19. d,e) FE‐SEM images of ECA 37. f,g) FE‐SEM images and corresponding elemental (C, O, and Cl) maps of LiCl@ECA 19. h,i) FE‐SEM images and corresponding elemental (C, O, and Cl) maps of LiCl@ECA 37.

And we prepare the emulsion precursors using different oil‐water ratios (1:9 and 3:7) to optimize the internal structures and properties of the CNC‐based aerogels (ECAs). After freeze‐drying, ECA 19 (Figure S5, Supporting Information) and ECA 37 (Figure S6, Supporting Information) are obtained. The scanning electron microscope (SEM) provided detailed visualization of the microstructures of ECA 19 and ECA 37. During the freeze‐drying process, the water and oil phases of emulsions are entirely removed, allowing CNCs assembly into the aerogels with various morphologies through hydrogen‐bonding interactions.^[^ ^43^, ^44^ ^]^ Generally, emulsions with a higher proportion of the oil phase facilitate a more micro‐porous structure of the subsequent aerogel prepared using the emulsion template method, owing to more droplets. In this light, the inner area of ECA 19 primarily consists of large channels with sizes in the range of several hundred micrometers, with some channels being arranged in parallel (**Figure** **2**b). In contrast, the porous structure of ECA 37 consists of large pores with sizes in the range of several hundred micrometers (Figure 2d). Zooming in further on one of the large pores, one observes that it is composed of many micrometers of small holes (Figure 2e). These small holes provide additional binding sites for dehumidifiers and increase the surface area available for water uptake. After that, ECA 19 and ECA 37 are decorated with LiCl using a freeze‐drying method to enhance their water uptake performance (Figures S7 and S8, Supporting Information). The Li^+^ and Cl^−^ ions quickly diffuse into the ECA networks and partially react within the ECA skeletons. During freezing and lyophilization, these two types of ions start to self‐aggregate into solid grains and become tightly embedded within the ECA networks, resulting in a slight contraction of these networks (Figure 2a). Notably, LiCl is uniformly distributed throughout the ECA networks (Figure 2f,h). Additionally, ECA 19 and ECA 37 retain their channel and porous structures, respectively, indicating that the combination of LiCl and ECA skeletons does not substantially affect the original morphologies of these aerogels. Moreover, the corresponding elemental maps also show that LiCl is evenly attached to the ECA skeletons, especially the Cl element derived from LiCl (Figure 2g,i).

### 2.2 Atmospheric Water Harvesting and Oozing Behavior of the LiCl@ECAs

**Figure 3:**
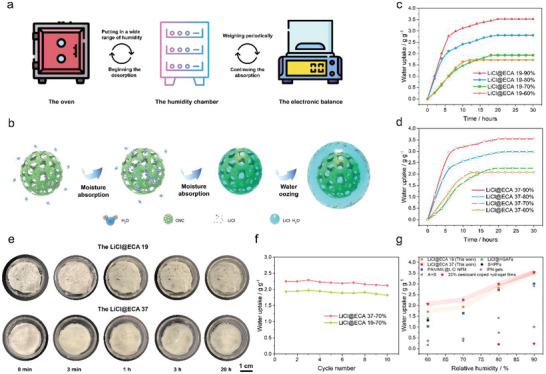
Evaluation of LiCl@ECAs’ moisture capturing capacity. a) Schematic diagram of LiCl@ECAs in the water sorption and absorption–desorption test cycles’ tests with a stable temperature and different degrees of humidity. b) Diagrammatic depiction showcasing the mechanism of moisture sorption and water release from the air by LiCl@ECAs. c,d) Evaluation of moisture absorption performance of LiCl@ECA 19 and LiCl@ECA 37 at 25 °C under varying RH. e) Visual representation showcasing the dynamic process of water molecular absorption by LiCl@ECA 19 and LiCl@ECA 37 at 25 °C and 90% RH through digital images. f) Examination of the stability of moisture absorption and desorption cycling for LiCl@ECA 19 and LiCl@ECA 37 at 25 °C and 70% RH, with a desorption period of 12 h under 103 °C. g) Comparison of our work with other reported studies regarding the moisture absorption capacity at various humidity levels.^[^ ^21^, ^25^, ^30^, ^45^, ^47^, ^48^ ^]^

This study evaluates the moisture‐capturing capacity of LiCl@ECAs, as depicted in **Figure** **3**a. Moreover, the mechanism of moisture absorption and liquefied water infiltrating in LiCl@ECAs is depicted in Figure 3b. Together, these processes involve the following steps: i) Due to the presence of LiCl, the surfaces of ECAs facilitate the assimilation and condensation of water molecules, leading the transformation of LiCl@ECAs into LiCl@ECAs·(H_2_O)_x_; ii) With an increase of moisture, the LiCl@ECAs·(H_2_O)_x_ deliquesce into a concentrated LiCl solution, gradually diffusing inward along the hydrophilic skeletons of the CNCs; iii) The amount of the absorbed moisture increases; the resulting LiCl‐saturated solution further dilutes and is stored in the ECAs; iv) When there is still much moisture in the air, the ECAs become thoroughly wet with water, leading excess moisture to ooze out as liquid water.^[^ ^21^, ^45^ ^]^ In other words, the synergistic effect of their material characteristics and composition structure significantly affects the hygroscopic behavior of LiCl@ECAs.

Furthermore, the moisture absorption properties of LiCl@ECAs are examined in this study using gravimetric measurements at 25 °C and varying relative humidity (RH) three conditions. Figure 3c illustrates the LiCl@ECA 19′s water uptaking capacities at various RH levels: 1.72 g/g (60% RH), 1.93 g/g (70% RH), 2.81 g/g (80% RH), and 3.52 g/g (90% RH). And LiCl@ECA 37 displays moisture sorption capacities at 60%, 70%, 80%, and 90% RH, measuring 2.07 g/g, 2.26 g/g, 2.99 g/g, and 3.54 g/g, respectively (Figure 3d). LiCl@ECA 19 and LiCl@ECA 37 are tested for their water sorption capacities at different RH levels of 60%, 70%, 80%, and 90% over an initial 8 h period. LiCl@ECA 19 demonstrates sorption capacities of 59.8%, 66.8%, 79.7%, and 84.9% of the premier amount, respectively. In contrast, LiCl@ECA 37 exhibits sorption capacities of 80.1%, 62.3%, 91.6%, and 88.1% of the initial amount, respectively. This observation aligns with the first three stages of the above‐mentioned mechanism. These results indicate that LiCl@ECAs can capture more water vapor from an ambient condition with higher humidity levels at a constant temperature, as more amounts of moisture can concomitantly interact with the active sites on LiCl@ECAs. Without changing the external environment, the equilibrium sorption capacity depends on the content of hygroscopic unit and the number of hydrophilic functional groups.^[^ ^46^ ^]^ Compared to LiCl@ECA 19, LiCl@ECA 37 exhibits higher saturated absorption capacities at 25 °C and 60%, 70%, and 80% RH levels. This can be attributed to the presence of numerous large pores and small holes, which increase the amount of LiCl and expose more hydrophilic oxygen‐containing functional groups for water absorbing. Interestingly, at 90% RH, the saturated adsorption moisture of the two samples is similar, as LiCl@ECAs can adsorb a sufficient amount of water molecules at this RH level.

Moreover, we monitor the surface changes of the studied samples during this period using a camera (Figure 3e). In turn, we find that initially, the surfaces are dry, rigid, and brittle, being primarily influenced by the presence of LiCl particles bound to the aerogel skeleton. After three minutes, the surfaces of the samples start to become wet, indicating moisture absorption from the air. After 3 h, a small quantity of liquid water appeared in the centers of the samples, particularly on the surface of LiCl@ECA 37. This is because the outer areas of these aerogels are denser than their middle areas, resulting in a higher moisture absorption rate in the outer areas. As a result, the water gradually diffuses to the middle areas, leading to wetting faster in the middle than in the outside areas. After 20 h, both the samples exhibit a water oozing phenomenon, demonstrating good hygroscopicity and the ability to capture a significant amount of water from moist air. Furthermore, the translucence observed in both samples confirms their capability to trap moisture from the atmosphere and maintain it in a liquid state. Further, the stability of both LiCl@ECA 19 and LiCl@ECA 37 during 10 cycles at 25 °C and 70% RH is demonstrated in Figure 3f. After 10 cycles, LiCl@ECA 19 exhibits a little reduction in water uptake capacity, reaching 94.3% (1.82 g/g) of its premier ability (1.93 g/g). And LiCl@ECA 37 retains its water uptake at 94.2% (2.12 g/g) of its original ability (2.25 g/g). Additionally, the pores and channels in the LiCl@ECAs decelerate their loss of LiCl, enhancing their long‐term stability. Furthermore, compared to other recorded desiccant materials, LiCl@ECAs demonstrate outstanding moisture absorption capacities over a wide range of RH levels (Figure 3g).^[^ ^21^, ^25^, ^30^, ^45^, ^47^, ^48^ ^]^

### 2.3 In situ Hygroscopic Monitoring

The local impedance of a LiCl@ECA changes according to its specific degree of moisture absorption. In this paper, the relative electrical conductivity primarily represents the impedance of the LiCl@ECAs under study. The working principle of the EIT system used in the in situ monitoring of the hygroscopic process under study is shown in Figure 1a. The LiCl@ECAs are equipped with 32 electrodes positioned around their boundaries. An electric current is injected into the LiCl@ECAs starting from the first and second electrodes, generating an electric field that creates an electric potential distribution over the LiCl@ECAs. The EIT system records the current and voltage values corresponding to each electrode. Subsequently, we switch to the next adjacent pair of electrodes for the current injection. After that, we measure the characteristic values again based on the new electric potential distribution. We repeat this process several times until the integrated device completes the current injection by the last two electrodes (Figure S9, Supporting Information).

**Figure 4:**
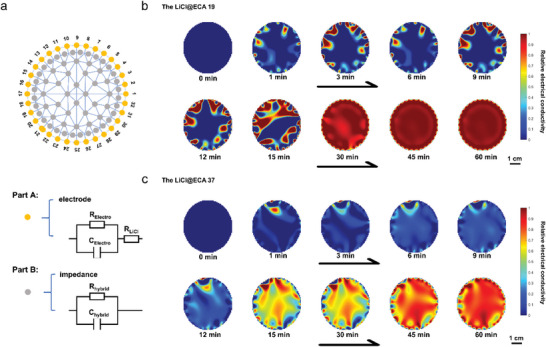
In situ monitoring of the hygroscopic process of LiCl@ECAs. Visualization of the hygroscopic process of LiCl@ECAs in an open environment at 25 °C and 60% RH through reconstructed EIT images captured throughout 1 h. a) Schematic diagram showing the devices in order to reconstruct the conductivity distribution based on pillar electrodes at the boundary of the wall used to monitor the LiCl@ECAs (voltage measurements were taken at every electrode for each current injection). b) The LiCl@ECA 19. c) The LiCl@ECA 37. The color bar represents the relative electrical conductivity value. For the purpose of quantitative comparison, we applied normalization to the corresponding eigenvalue, ensuring a uniform scale.

In the graphical representation of **Figure** **4**a, the impedance data recorded during the abovementioned process are utilized as input for a reconstruction algorithm that estimates the relative conductivity distribution within the LiCl@ECAs. This algorithm draws inspiration from the finite element analysis that discretizes the domain of LiCl@ECAs into a finite number of elements and nodes, employing the Tikhonov method. This method facilitates designing an impedance network model with a triangular, arbitrarily shaped mesh and a conductivity distribution.^[^ ^49^ ^]^ In this regard, Part A is the electrode–LiCl@ECAs model that describes the contact impedance. At the same time, Part B constitutes the basic unit of the impedance network in the LiCl@ECAs, describing the impedance characteristics of the composite hygroscopic unit at a specific location. Based on the EIT system, the above reconstruction algorithm dynamically displays the moisture absorption process of the LiCl@ECAs.

The EIT device tests the LiCl@ECAs at 25 °C and 60% RH. The reconstructed images indicating relative conductivity are demonstrated in Figure 4b,c. Initially, the images of the two studied samples are similar since they are in a dry state. However, after 1 min, the edge of LiCl@ECA 19 begins to absorb moisture while its internal space remains unchanged. Within the first 15 min, the LiCl@ECA 19 shows a hygroscopic trend from its edge to its interior (Figure 4b). Generally, CNCs possess many surface hydroxyl groups and are highly hydrophilic. As a result, the liquid water generated by hygroscopicity is transported along the CNCs’ network to the inner part of LiCl@ECA 19 through capillary force, thereby being stored throughout ECA 19. In contrast, both the edges and the interior of LiCl@ECA 37 start absorbing moisture simultaneously. The discrepancy can be explained by the higher number of moisture absorption sites and less compact internal structure of LiCl@ECA 37 in comparison to LiCl@ECA 19, which increases the former's probability of contact with the external environment. At the twelfth minute, LiCl@ECA 37 exhibited an average relative conductivity of 0.41 S m^−1^, which decreased to 0.16 S m^−1^ at the fifteenth minute. In this study, the continuous fiber network and LiCl modify their local or overall relative conductivity through water adsorption, while the EIT system generates a map of moisture absorption changes. Simultaneously, the relative conductivity serves as an average representation of moisture content across all vertical directions at the specific location. Consequently, significant changes in relative conductivity during this period indicate substantial variations in moisture absorption for LiCl@ECA 37. Besides, the test results regarding temperature and resistance for LiCl@ECAs are consistent with the concomitant results of the EIT (Figures S10–S12, Supporting Information).

Interestingly, the abovementioned process can be divided into two stages (Movies S1 and S2, Supporting Information). During the first stage, both LiCl@ECA 19 and LiCl@ECA 37 gradually absorb moisture from the air. Then, after 30–45 min, the hygroscopicity of both samples rapidly increases, displaying their increasing absorption kinetics, aligning with the conclusions of the moisture absorption experiments in Figure 3c,d. Furthermore, at 60 min, the color of LiCl@ECA 37 is lighter than that of LiCl@ECA 19, indicating that the relative conductivity of the former is lower, in correspondence with its lower concentration. This confirms that the moisture absorption capacity of LiCl@ECA 37 is higher, thus corroborating the distinct hygroscopic evolutions of the studied samples. Given the essential role of geometric and structural features in hygroscopic materials, the samples are thoroughly examined through N_2_ adsorption measurements to provide systematic insights. According to Barrett–Joiner–Halenda model, the corresponding pore size results show that the average pore sizes of the ECA 19 and the ECA 37 are 8.7241 nm and 3.6449 nm, respectively (Table S1, Supporting Information). After loading LiCl, the average pore sizes of LiCl@ECA 19 and LiCl@ECA 37 changed to 6.9717 and 6.1731 nm, respectively. The average pore sizes mentioned above exceed the kinetic diameter (≈0.29 nm) of a water molecule,^[^ ^50^ ^]^ which facilitates the absorption of water molecules and transportation of liquefied water. In general, mesoporous materials with pore sizes in the range of 2–50 nanometers are often considered favorable for water vapor sorption. These sizes allow for sufficient surface area and accessibility while facilitating efficient adsorption processes. The results show that the pore diameters of all samples are concentrated ≈1–6 nm (Figure S13, Supporting Information), belonging to 2–50 nm, which is beneficial for water vapor sorption. In this study, we synthesized two different types of hygroscopic materials, LiCl@ECA 19 and LiCl@ECA 37, with varying pore size distributions. By combining qualitative results from moisture absorption performance, SEM, EIT, and BET measurements' quantitative results, preliminary insights can be derived regarding the relationship between pore size and moisture absorption kinetics. It indicates that smaller pore sizes tend to restrict the movement of water molecules, leading to slower absorption kinetics. Conversely, larger pore sizes provide more pathways for water molecules to penetrate the material, resulting in faster absorption kinetics. Therefore, the pore size of the composites may significantly impact the kinetics of their high moisture absorption.

### 2.4 Validation of the Hygroscopic Mechanisms of LiCl@ECAs

**Figure 5:**
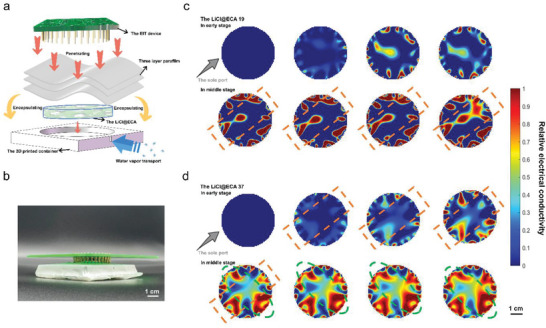
Verification of the EIT technique for real‐time detection of the water uptake process and the hygroscopic mechanisms of LiCl@ECAs. a) Schematic diagram outlining the steps involved in the unidirectional moisture absorption measurement using the EIT system. b) Picture depicting the setup of the EIT system with parafilm‐encapsulated LiCl@ECAs, utilized in the unidirectional hygroscopic experiment. We reconstruct the EIT images regarding the hygroscopic process of LiCl@ECAs under the condition that one sole channel would remain connected to the air. A testing temperature of 25 °C, a RH of 60%, and a 2 h test duration is maintained throughout the experiment. c) The LiCl@ECA 19. d) The LiCl@ECA 37. The color bar represents the relative electrical conductivity value. Normalization was performed on the corresponding eigenvalue using a uniform scale to facilitate quantitative comparison.

This study's experimental results and theoretical analysis elucidate the enhanced hygroscopic activities of the LiCl@ECAs, resulting from their hydrophilic cellulose network and the amplified number of water sorption areas. Here, we specifically focus on evaluating the unidirectional moisture absorption behavior of the LiCl@ECAs. To assess the same, the LiCl@ECAs are placed in a three dimensional printing model with only one port, and the EIT device is utilized to monitor the entire assessment process for 2 h (**Figure** **5**a,b; Figure S14, Supporting Information).

Figure 5c demonstrates that LiCl@ECA 19 continues to exhibit moisture absorption at its edges during the early stage of the assessment. This shows that even with a single water contact channel, hydrogen bonding, and capillary force allow the rapid transport and storage of adsorbed liquid water within this aerogel. As the process progresses to the middle stage, LiCl@ECA 19 demonstrates oriented water diffusion behavior along the sole port (indicated by the orange dotted area in Figure 5c). This observation suggests that LiCl@ECA 19, with only a single channel connecting to the outside atmosphere, exhibits directional moisture absorption behavior in addition to absorbing moisture from the external area into its internal structure. Consequently, it results in an anisotropic moisture absorption state. On the other hand, due to its higher water‐absorbing kinetics, LiCl@ECA 37 already exhibits directional moisture absorption behavior in the early assessment stage (also indicated by the orange dotted area). In the middle stage, because of the rise moisture absorption degree and the presence of liquid water, the different areas of water storage within LiCl@ECA 37 gradually integrate, showing a tendency to move inward (Figure 5d, the green dotted area), resulting in an isotropic moisture absorption state. Furthermore, throughout the assessment process, LiCl@ECA 37 demonstrates a more substantial water adsorption capacity than LiCl@ECA 19, indicating that the absorption sites and diffusion pathways of aerogels are essential factors that influence their moisture absorption capacities and other relevant properties (Movies S3 and S4, Supporting Information). Interestingly, affected by these aerogels’ material properties and composition structure, the amount of their moisture absorption and the direction of their moisture diffusion demonstrate intrinsic self‐selective behavior.

## 3 Conclusion

Despite significant advancements in AWH technologies in recent years, there remains a need for comprehensive research on the hygroscopic behavior of AWH and the mechanisms associated with such behavior. This limitation hinders scholarly progress toward understanding the fundamental potentials of matrices and sorbents used in AWH systems. However, understanding the hygroscopic kinetics of AWH concerning matrices and sorbents is crucial for unlocking the full hygroscopic potential of AWH technologies. This involves exploring novel in situ techniques for detecting the water absorption process, which can serve as a shortcut to establishing or validating the underlying mechanisms of AWH systems. In this respect, our study focuses on producing a dynamic visualization of moisture diffusion in hygroscopic aerogels using an EIT technique with a reconstruction algorithm. Using a LiCl‐cellulose‐based aerogel as a typical hygroscopic system, our study findings reveal that water molecules undergo absorption and condensation on the surfaces of aerogels, and collected liquid water is transported along their polymer skeletons. By systematically characterizing the different structures of LiCl@ECAs at varying RHs, we demonstrate that one of the key factors contributing to high moisture absorption performance of aerogels is their pore sizes; the salience of these factors is also revealed in some other reports.^[^ ^51^, ^52^ ^]^ In particular, we develop a generalized EIT monitoring system to conduct a qualitative study of the water sorption processes associated with hygroscopic materials having different microstructures; further, we investigate the underlying hygroscopic kinetics of these materials. The results of our study reveal a robust self‐selective behavior by these hygroscopic gels in terms of the diffusion path of absorbed moisture and the distribution of stored moisture. Notably, hydrogen bonding and capillary force facilitate transporting and storing adsorbed moisture within the aerogels. This indicates that the polymer skeleton of an aerogel acts as a high‐speed water transport pathway, expediting the sorption kinetics and enhancing water absorption.^[^ ^9^, ^45^ ^]^ These detections make a substantial contribution to study how hygroscopic mechanisms operate in LiCl‐cellulose‐based aerogels.

Our in situ EIT monitoring system offers real‐time visual monitoring for ion transport monitoring. The EIT method distinguishes its characterizations from the traditional characterizations in the previous related studies regarding variations in raw materials, device structure, and working principles. The utilization of the EIT monitoring system simplifies the characterization process by eliminating the limitations in the scope of detection. Additionally, this technology is cost‐effective, portable, and environment‐friendly, delivering significant advantages compared to traditional methods. These advantages highlight its potential for application in future studies in the field of AWH. Besides, EIT is well‐suited for characterizing materials with non‐uniform internal conductivity. Its application conditions necessitate the tested object to possess a certain level of conductivity and fall within the impedance range of the measuring equipment. To be more specific, the imaging principle of EIT is specifically derived from the varying conductivity of the measured object. This indicates that the EIT system is sensitive to changes in conductivity rather than the moisture content within the components. In other words, in the context of in situ imaging technology using EIT systems, the accuracy of the results improves when there is a more noticeable change in the conductivity of hygroscopic materials. Thus, through the utilization of water adsorption on various media to induce conductivity changes either overall or locally, it becomes feasible to create a mapping of the hygroscopic variations. Moreover, researchers have verified the feasibility of the mapping relationship between the force and the reconstructed conductivity of the hydrogel (without salt) based on the EIT strategy.^[^ ^8^, ^9^, ^53^ ^]^ Therefore, composite hygroscopic sorbents with conductivity have the potential to be visualized using the EIT system when there are observable changes in their conductivity, such as during drying and moisture absorption.

Indeed, EIT can help researchers better explain the unexplored characteristics, behaviors, and mechanisms of hygroscopic materials. Significantly, addressing the issues of limited spatial resolution and sensitivity to noise can further optimize the performance and facilitate the simultaneous practical applications of EIT. At present, the two dimensional (2D) water transport under various conditions has been extensively investigated and validated using the EIT system. Apart from water transport within the plane, the water transport along the thickness direction is equally significant in the water sorption process. The conductivity distribution can be measured by modifying the distribution of the measuring electrodes to three dimensional (3D), thus can be used to image the distribution of moisture in each layer of hygroscopic materials. Besides, 3D‐EIT systems have proven feasibility in agriculture, engineering, and other fields.^[^ ^54^, ^55^, ^56^ ^]^ Building upon the verification of the EIT system's capability to monitor real‐time moisture transport of materials on a 2D scale, our future focus will involve upgrading algorithms and hardware to study water transport along the thickness direction, which providing a more comprehensive assessment of the water absorption condition of hygroscopic materials.

In summary, we develop a generalized EIT technique with a reconstruction algorithm to investigate the moisture sorption mechanisms of hygroscopic aerogels. While this work focuses on a specific material system of LiCl‐cellulose‐based aerogels, the EIT monitoring system can be extended to the purposes of understanding, studying, and designing different types of AWH materials, such as hygroscopic hydrogels, foams, or sponges. Moreover, the approach devised in this study presents a novel outlook on how each component of a hygroscopic gel dynamically responds while absorbing moisture, highlighting the crucial role of the synergy between sorbents and segments of the polymer matrix in this process. Notably, our approach foregrounds a pioneering and versatile means of in situ and dynamic hygroscopic monitoring, providing valuable insights for future studies on and the designs of various sorption‐based materials associated with the field of AWH.

## 4 Experimental Section

### Materials

The cellulose nanocrystals were obtained from Zhongshan Naxiansi New Technology Co., Ltd. (China). Vinyl acetate and methylcellulose were obtained from Shanghai Macklin Biochemical Co., Ltd. (China). LiCl was purchased from Scharlab. The reagents and chemicals utilized in this study were employed as received without any additional purification procedures.

### Preparation of CNCs‐Based Aerogels

The CNCs suspension was sonicated (800 W and 20 min; ATPIO‐1250DN) and then diluted to a concentration of 0.15%. Subsequently, the preparation of the strengthened CNCs included freezing the CNCs suspension using liquid nitrogen and freeze‐drying after that (24 h; LC‐10N‐50A). In a separate step, 300 mg of CNCs and 30 mg of methylcellulose were added to a mixture of 15 mL vinyl acetate and deionized water (with oil/water ratios of 1:9 and 3:7). This mixture was then subjected to sonication to form emulsions (800 W and 30 minutes or 30 min). In this process, methylcellulose acted as an auxiliary emulsifier. Finally, the CNC‐based aerogels were prepared by subjecting them to freeze‐drying for 12 h.

### Preparation of LiCl@ECAs

The LiCl@ECAs were obtained by immersing the CNC‐based aerogels in an 8 mL solution of 10 wt.% LiCl for ≈12 h and subjecting them to freeze‐drying for 12 h.

### Characterization of Materials

The morphology and microstructure of studied samples were characterized using SEM (JSM‐6390) and FE‐SEM (Regulus 8100). The element distribution in the cross‐section of studied samples was characterized using an energy‐dispersive spectrometer (EDS). Using a thermocouple (UNI‐T321), temperature measurements were taken for the outer and inner sections of LiCl@ECAs in an environment with the following specifications: 25 °C and 60% RH. The resistance of LiCl@ECAs in both dry and saturated hygroscopic states at 25 °C and 60% RH was measured using the source measure unit (Keithley 2450). The pore sizes of all samples are determined by N_2_ adsorption isotherms (Belsorp‐miniX, MicrotracBEL).

### Moisture Sorption Measurement

The samples were housed in temperature and humidity‐controlled equipment (MHU‐150L, TERCHY) to evaluate their moisture sorption capacity and the stability of absorption‐desorption cycles. After that, the moisture sorption tests were carried out at a constant temperature of 25 °C while subjecting the samples to varying humidity levels, including 60%, 70%, 80%, and 90% RH. Before conducting tests, studied samples were dried in an oven at 103 °C until a consistent weight was attained. Afterward, studied samples were placed in temperature and humidity‐controlled equipment with consistent temperature and RH to ensure environmental stability. After that, at regular intervals of 2 h, studied samples were extracted and swiftly weighed using an electronic balance with a precision of 0.0001 g. The studied samples were then exposed to a stable temperature of 25 °C and a RH of 70% for 20 h to enable moisture absorption, thereby finalizing the absorption‐desorption cycle. Subsequently, the samples were placed in an oven and subjected to desorption at 103 °C for 12 h. This process was repeated ten times.

The calculation of water sorption for the samples was performed using Equation 1:

(1) $$Water\;sorption=\frac{W_{w}}{W_{s}}$$

where *W*_w_ was the weight of the moisture absorbed by the samples and *W*_s_ was the weight of the samples before moisture absorption.

### Fundamentals of the EIT device

The LiCl@ECAs were placed at 25 °C and 60% RH, with apparent changes in conductivity distribution δ(*x*, *y*) during the experiments. These kinds of changes were acquired at the boundary of the sample through an EIT system, consisting of a 32‐electrode array, a four‐terminal impedance measurement device, and the software on a computer. The relationship between the measured impedance matrix **Z** and δ(*x*, *y*) refers to the following formula^[^ ^57^ ^]^:

(2) $${Z|}_{AB}^{CD}=-\frac{1}{I^{2}}{\;\int \int \;}_{\Omega }\delta \left(x,y\right)\nabla \Phi_{\mathrm{AB}}\left(x,y\right)\nabla \Phi_{\mathrm{CD}}\left(x,y\right)d\Omega$$

where Ω is the region of interest (ROI) surrounded by the electrode array. ${Z|}_{AB}^{CD}$ is the transimpedance measured by the adjacent electrode‐pair *C*‐*D*, when an AC source *I* is applied at the adjacent electrode‐pair *A*‐*B* (as the adjacent electrode‐pair 1–2 and 3–4 in Figure 4a). Φ_AB_ and Φ_CD_ denote the electric potential when the source is applied at *A*‐*B* and *C*‐*D*, respectively.

The formula 2 can be rewritten according to the finite element analysis (FEA):

(3) $${Z|}_{AB}^{CD}=-\frac{1}{I^{2}}\sum_{n\;=\;1}^{N}\delta \left(n\right)\nabla \Phi_{\mathrm{AB}}\left(n\right)\nabla \Phi_{\mathrm{CD}}\left(n\right)$$

where Ω is divided into *N* meshes. δ, Φ_AB_, and Φ_CD_ can be regarded as fixed values in each mesh. To simplify the calculation process, the changes in Φ_AB_ and Φ_CD_ are ignored according to the Taylor decomposition. Thus, when considering all the measured impedances, the linear model of EIT is obtained:

(4) $$S_{U\times N}\;g_{N\times 1}=\lambda_{U\times 1}$$

where **S**_*U* × *N*_, **g**_*N* × 1_, and **λ**_*U* × 1_ is normalized sensitivity matrix, conductivity changes (Δ**δ**), and impedance changes (Δ**Z**), respectively. *U* is the number of measured impedance changes, which can be calculated through $U\;=\frac{X\times (X-3)}{2}\;$. *X* denotes the number of the electrode‐pairs and is 32 in this research. The component *S*(*u*, *n*) of the normalized sensitivity matrix can be expressed as:

(5) $$S\left(u,n\right)\propto \nabla \Phi_{u-\mathrm{ex}}\left(n\right)\nabla \Phi_{u-\mathrm{mea}}\left(n\right)$$

where u − ex and u − mea are the excitation and measurement electrode‐pair, respectively, corresponding to the *u* ^th^ Δ**Z**.

During the experiments, **g**_*N* × 1_ observed as the gray levels of pixels for the purpose of visualization is the only unknown value, while **S**_*U* × *N*_ can be obtained through FEA simulation. The calculation process of the Formula 4 is a typical inverse problem in mathematics, which can be solved through the Tikhonov method adopted by us^[^ ^49^, ^58^ ^]^:

(6) $$g\;={\left(S^{T}S+\mu I\right)}^{-1}\;S^{T}\lambda$$

where **S** ^*T*^ is the transpose matrix of **S**_*U* × *N*_. **I** is the identity matrix with N×N components. μ donates the positive regularization parameter.

The LiCl@ECAs were placed inside a 3D‐printed prototype and sealed using a parafilm (PM‐999) so that the EIT could measure the unidirectional moisture absorption. The mold for the concomitant model was created using a light‐curing 3D printer (CREALITY, LD‐002H) and photosensitive resin (QIFENG, 282 PRO). The sensor of 32 electrodes was able to penetrate the parafilm and be inserted at the edge of the sample. Following the abovementioned setup and procedure, the needle pierced the parafilm at the designated site for the sole pole when the EIT system was turned on.

The calculated Equation 7 of the relative electrical conductivity (σ) is as follows.

(7) $$\sigma \;=\frac{1}{Z}$$

where *Z* is the measured electrical impedance.

## Conflict of Interest

The authors declare no conflict of interest.

## Supporting information

Supporting Information

Supplemental Movie 1

Supplemental Movie 2

Supplemental Movie 3

Supplemental Movie 4

## Data Availability

The data that support the findings of this study are available from the corresponding author upon reasonable request.
