# Supplementary material for: In situ Electrical Impedance Tomography for Visualizing Water Transportation in Hygroscopic Aerogels
Source: Adv Sci (Weinh). 2024 May 14;11(29):2402676. doi: 10.1002/advs.202402676 (PMC11304325; doi:10.1002/advs.202402676)
Supplement: Supplementary file 1 — Supporting Information [file ADVS-11-2402676-s004.docx]

Supporting Information

**In situ Electrical Impedance Tomography for Visualizing Water Transportation in Hygroscopic Aerogels**

*Miao Tang^1^, Haosong Zhong^1^, Xupeng Lu^1^, Rongliang Yang^1^, Connie Kong Wai Lee^1^, Yexin Pan^1^, Yi Chen^1^, and Mitch Guijun Li^1^**

^1^Center for Smart Manufacturing, Division of Integrative Systems and Design, The Hong Kong University of Science and Technology, Clear Water Bay, Kowloon, Hong Kong, SAR 999077, China

*Email: mitchli@ieee.org


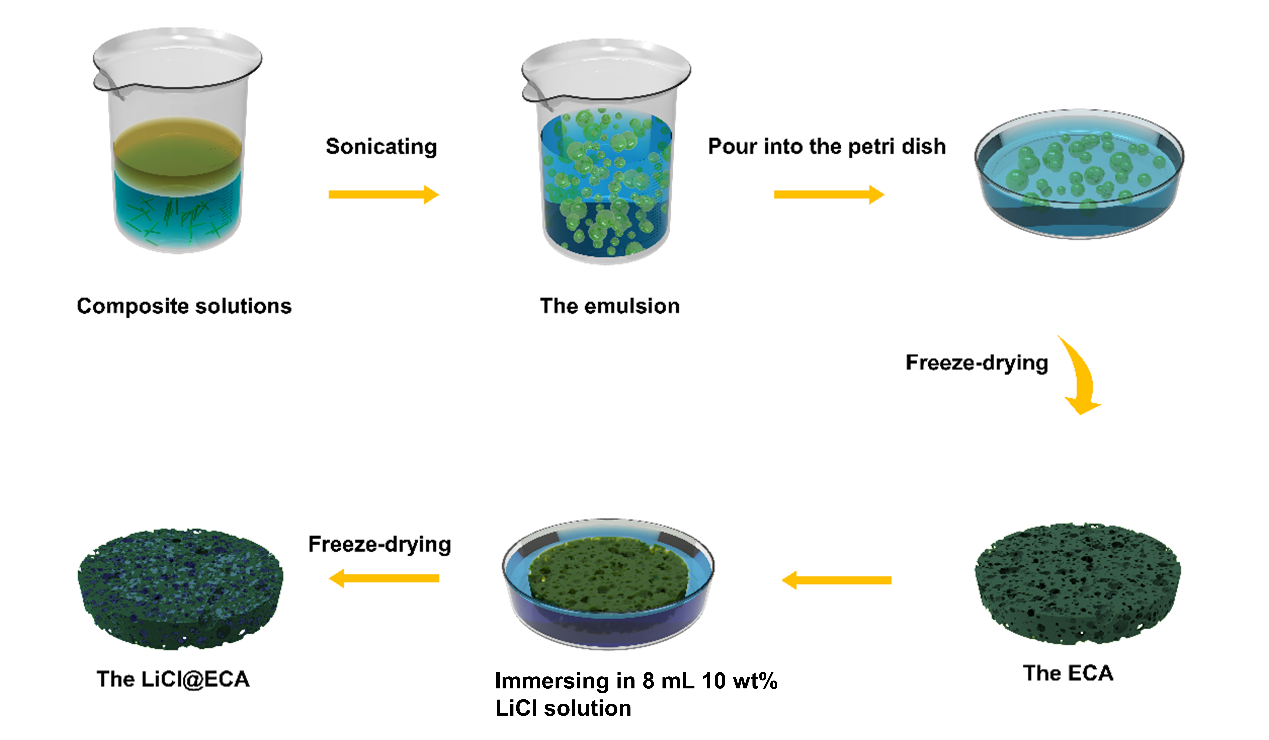


**Figure S1.** A schematic diagram of the process for fabricating the LiCl@ECA.


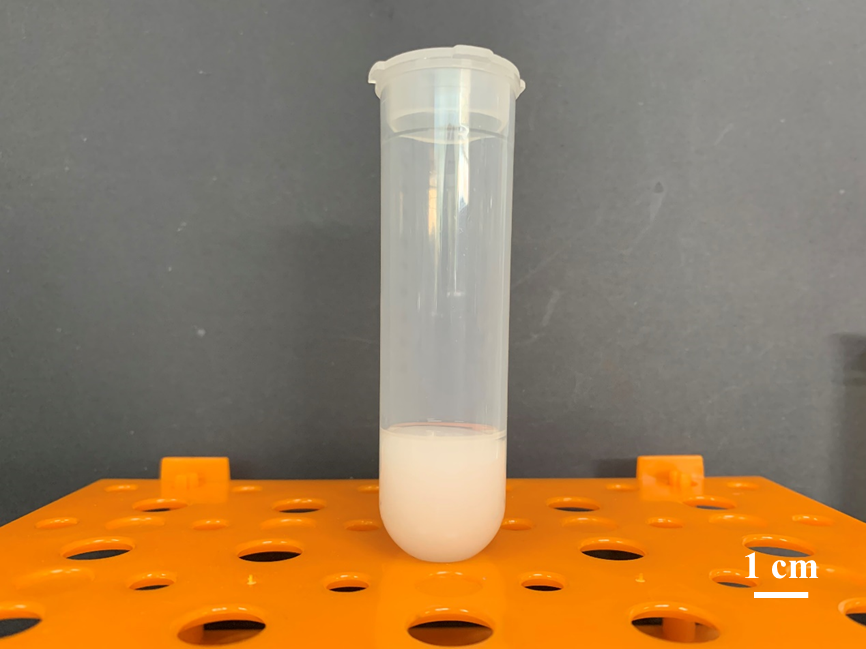


**Figure S2.** Digital photograph of the emulsion with 1:9 oil-water ratios.


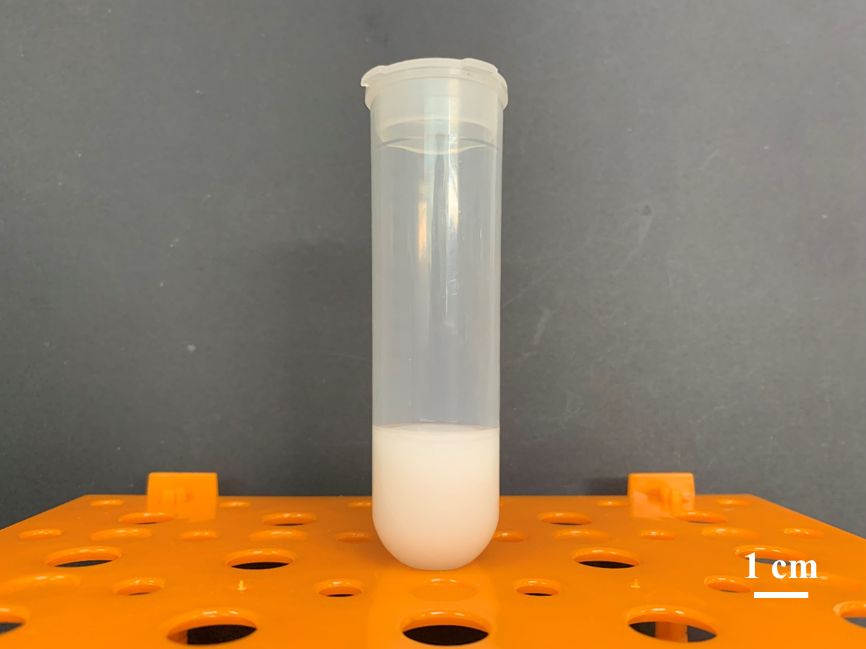


**Figure S3.** Digital photograph of the emulsion with 3:7 oil-water ratios.


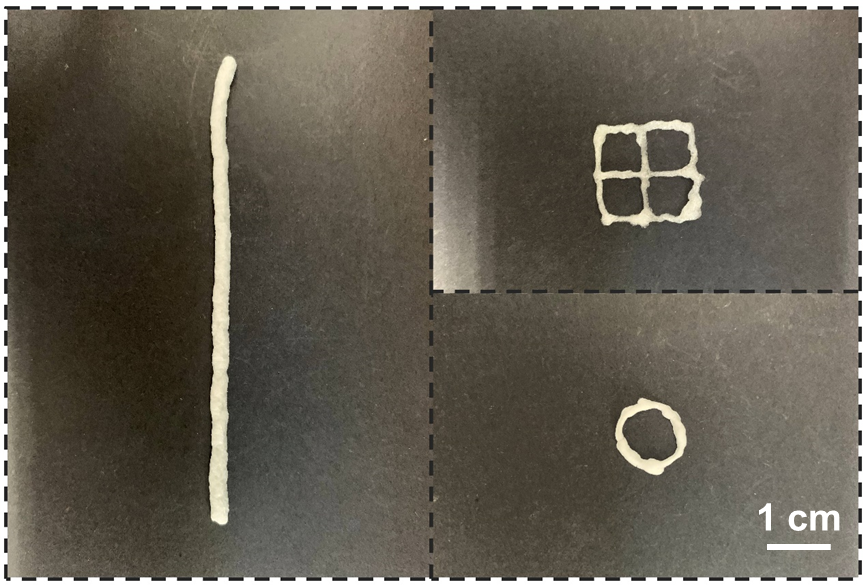


**Figure S4.** Digital photograph of the ECAs with different sizes and shapes.


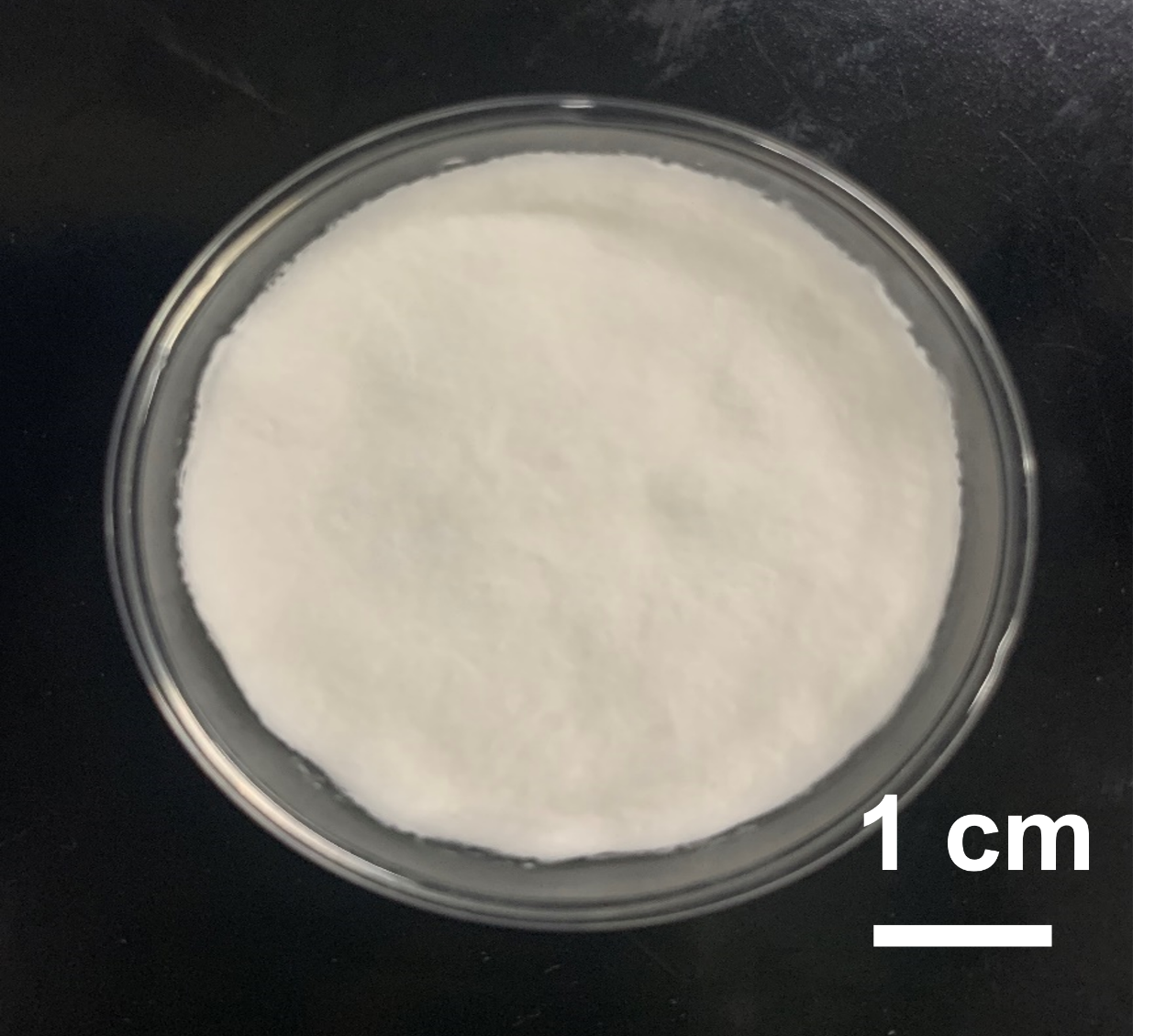


**Figure S5.** Digital photograph of the ECA 19.


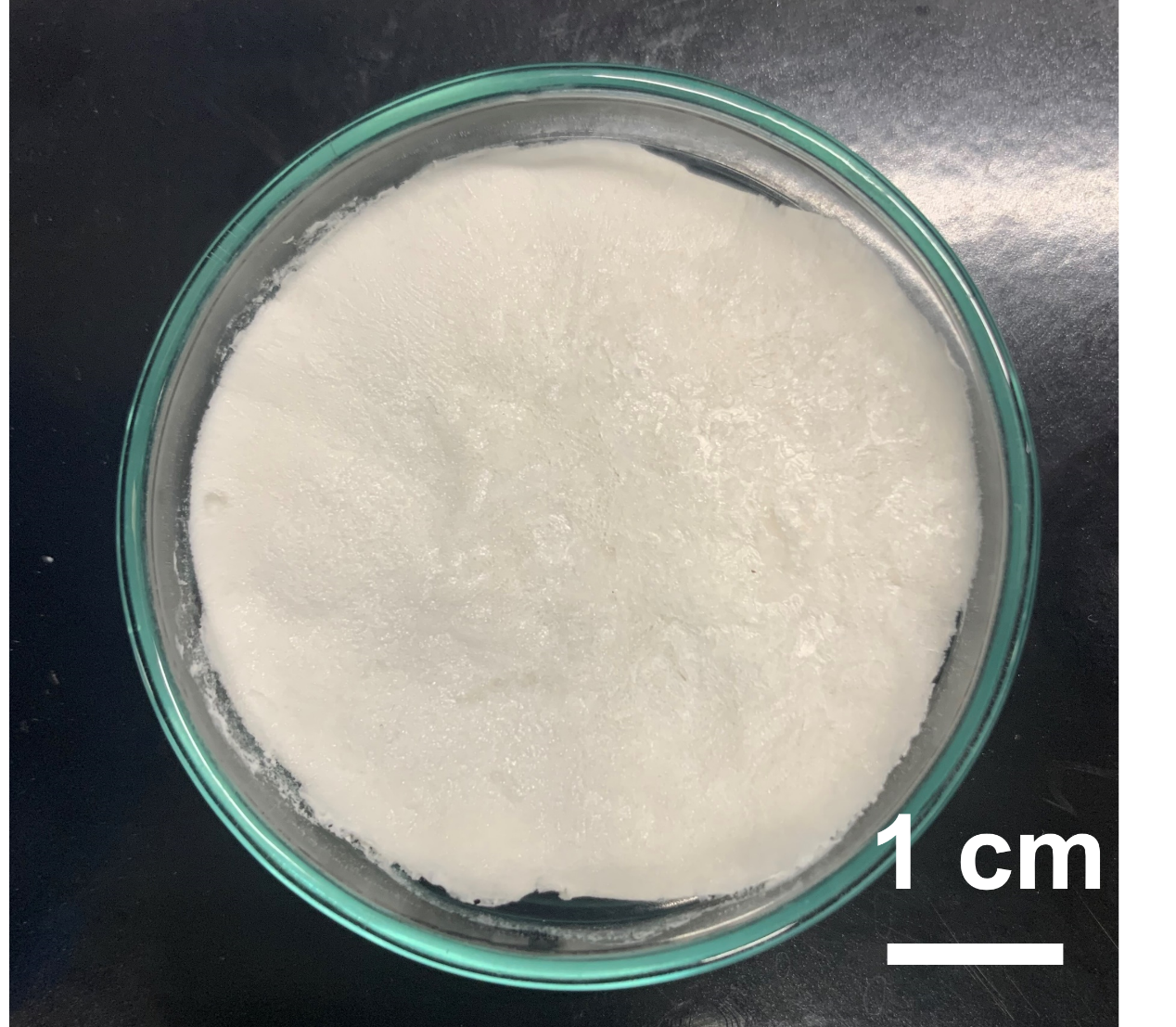


**Figure S6.** Digital photograph of the ECA 37.


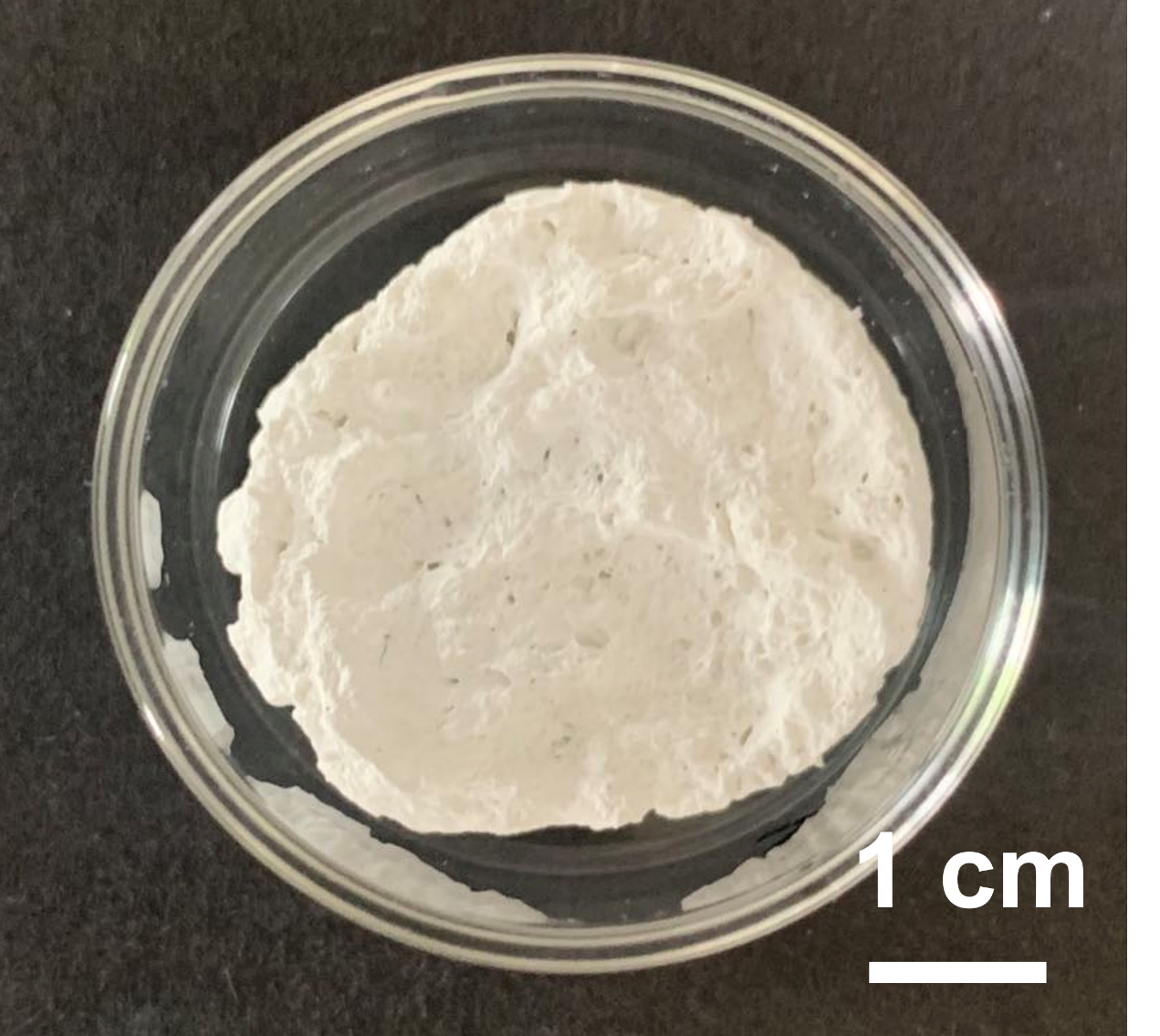


**Figure S7.** Digital photograph of the LiCl@ECA 19.


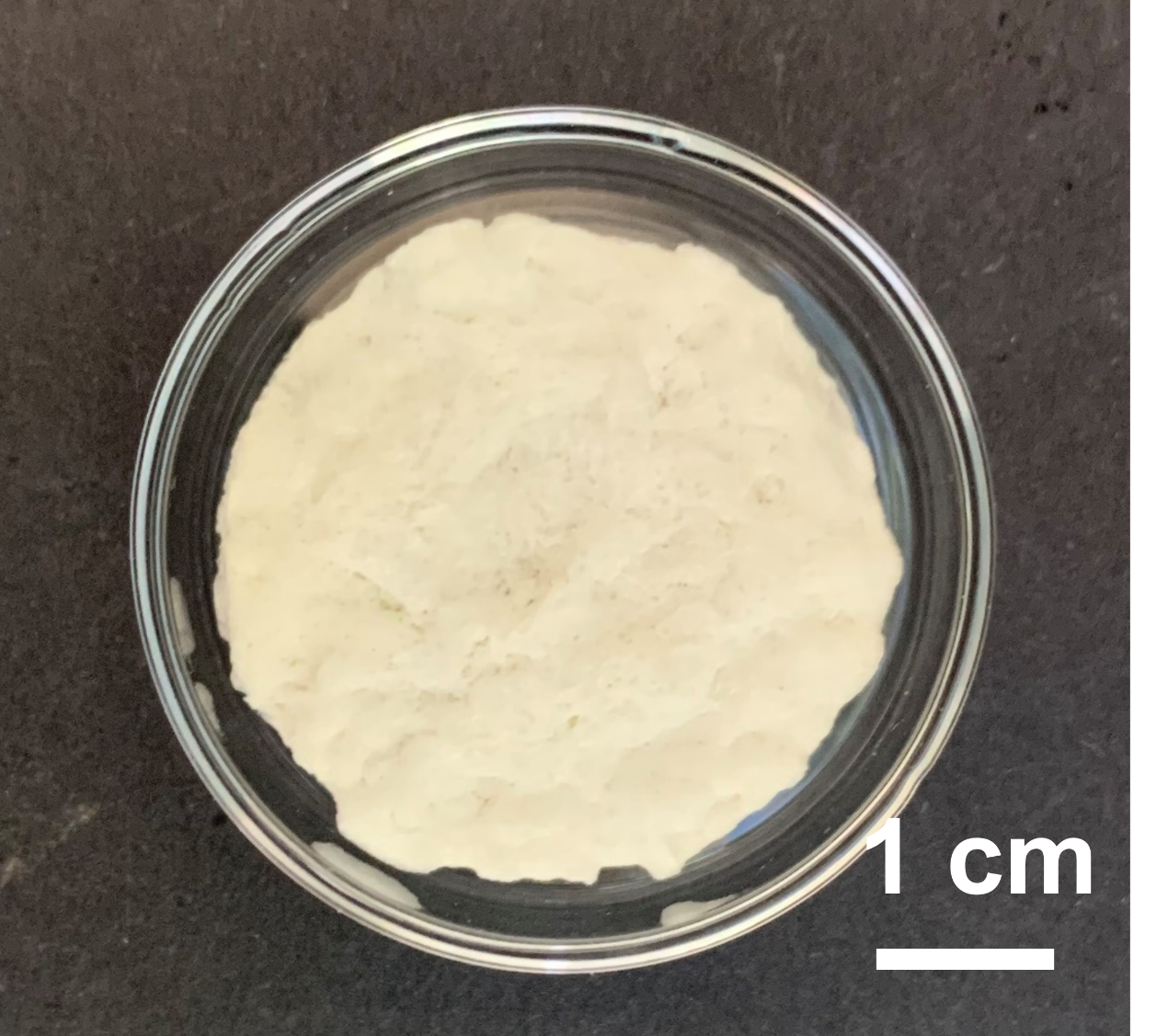


**Figure S8.** Digital photograph of the LiCl@ECA 37.


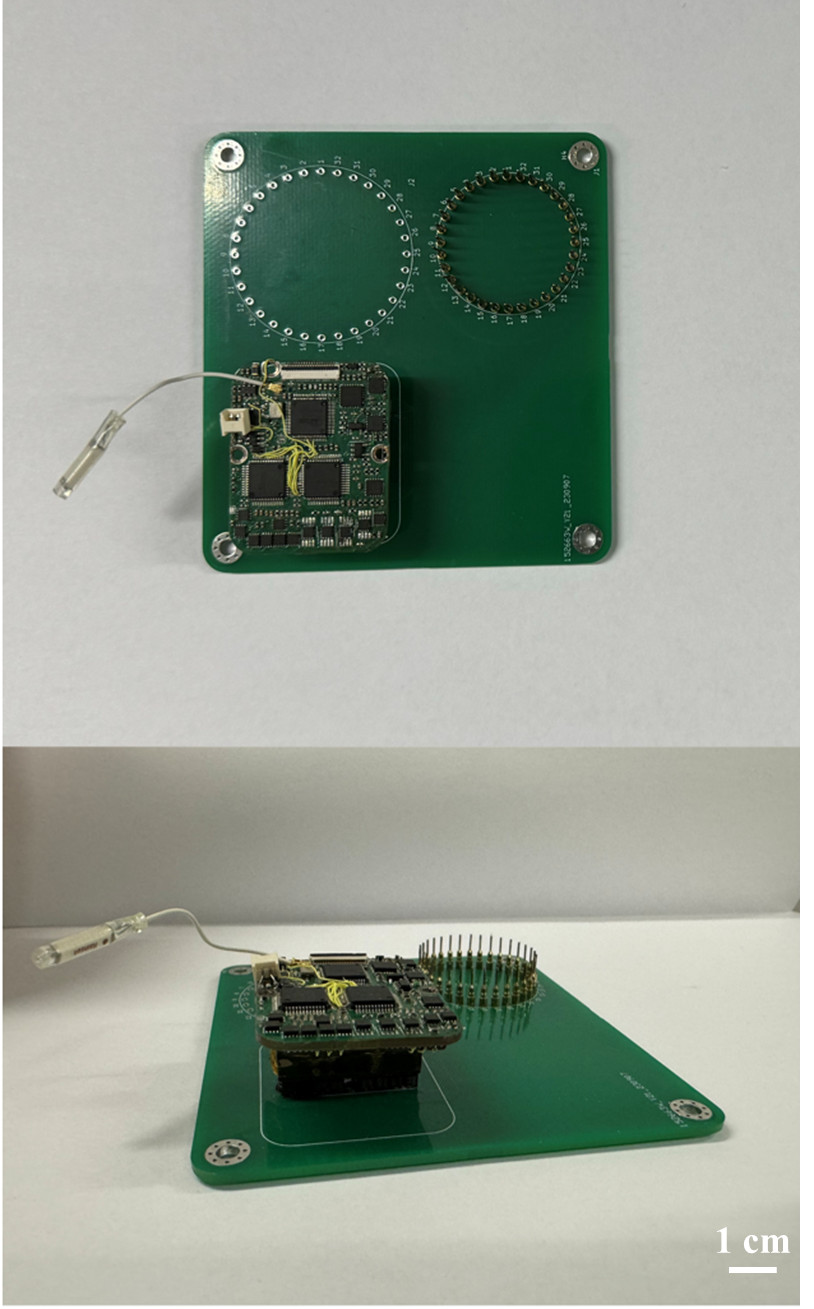


**Figure S9.** Photo of the EIT measurement device.

**Figure S10.** External and internal temperature variations of LiCl@ECA 19 at 25°C under 60% RH.

**Figure S11.** External and internal temperature variations of LiCl@ECA 37 at 25°C under 60% RH.

**Figure S12.** The diagram of LiCl@ECAs’ resistance under dry and saturated hygroscopic conditions at 25 °C under 60% RH.

**Figure S13.** Pore size distribution curves of the ECA 19, the ECA 37, the LiCl@ECA 19, and the LiCl@ECA 37, respectively.


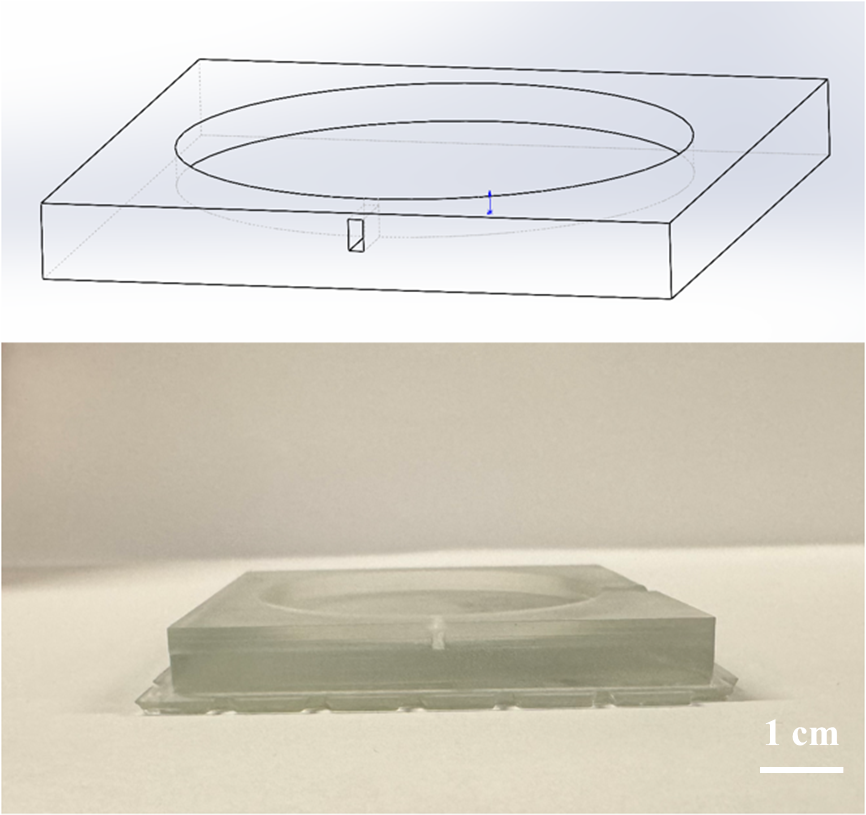


**Figure S14.** The device for unidirectional moisture absorption EIT measurement. (a) The designed model. (b) The model is fabricated with 3D printing.

**Table S1.** The pore sizes of all samples by N_2_ adsorption isotherms under Barrett-Joiner- Halenda model.

|  | Average pore diameter/nm | Median pore diameter/nm |
| --- | --- | --- |
| The ECA 19 | 8.7241 | 4.7605 |
| The ECA 37 | 3.6449 | 9.6257 |
| The LiCl@ECA 19 | 6.9717 | 66.129 |
| The LiCl@ECA 37 | 6.1731 | 16.154 |

**List of SI videos**

**Movie S1.** The EIT real-time image of the LiCl@ECA 19 for 1 hour in an open environment of 25 °C and 60% RH (mp4).

**Movie S2.** The EIT real-time image of the LiCl@ECA 37 for 1 hour in an open environment of 25 °C and 60% RH (mp4).

**Movie S3.** The EIT real-time image of the LiCl@ECA 19 for 2 hours in the unidirectional environment of 25 °C and 60% RH (mp4).

**Movie S4.** The EIT real-time image of the LiCl@ECA 37 for 2 hours in the unidirectional environment of 25 °C and 60% RH (mp4).
